# Bcl6 for identification of germinal centres in salivary gland biopsies in primary Sjögren's syndrome

**DOI:** 10.1111/odi.13276

**Published:** 2020-01-28

**Authors:** Uzma Nakshbandi, Erlin A. Haacke, Hendrika Bootsma, Arjan Vissink, Fred K. L. Spijkervet, Bert van der Vegt, Frans G. M. Kroese

**Affiliations:** ^1^ Department of Rheumatology and Clinical Immunology University of Groningen University Medical Center Groningen Groningen The Netherlands; ^2^ Department of Oral and Maxillofacial Surgery University of Groningen University Medical Center Groningen Groningen The Netherlands; ^3^ Department of Pathology and Medical Biology University of Groningen University Medical Center Groningen Groningen The Netherlands

**Keywords:** B‐cell hyperactivity, germinal centre, lymphoid organisation, primary Sjögren's syndrome

Histopathological assessment of salivary gland biopsies is an important element of the diagnostic workup of Sjögren's syndrome (SS) (Fox, 2017; Kroese, Haacke, & Bombardieri, 2018). Microscopic evaluation of salivary glands of primary SS (pSS) patients reveals characteristic periductal lymphocytic infiltrates (foci), which mainly consist of T and B lymphocytes, as well as a variety of non‐lymphoid cells, including dendritic cells and macrophages. Over time, these infiltrates may become organised to ectopic lymphoid tissue with T/B cell compartmentalisation, presence of CD21^+^ follicular dendritic cell (FDC) networks and high endothelial venules (Fisher et al., 2017; Kroese et al., 2014, 2018; Salomonsson et al., 2003). Germinal centres (GCs) are present within this ectopic lymphoid tissue in roughly one‐quarter of the salivary gland biopsies of pSS patients and their presence is associated with more severe disease compared to GC‐negative pSS patients (Risselada, Looije, Kruize, Bijlsma, & Roon, 2013). These glandular ectopic GCs express mRNA encoding for activation‐induced deaminase, an enzyme critical for the induction of somatic hypermutation and essential for the main function of GCs, the generation of high‐affinity memory B cells (Bombardieri et al., 2007; Le Pottier et al., 2009; Muramatsu et al., 2000).

Presence of GCs in biopsies taken for the diagnosis of pSS has been suggested to be a risk factor for lymphoma development (Nishishinya et al., 2015; Sène et al., 2018; Theander et al., 2011), a finding recently disputed by us (Haacke et al., 2017, 2019). Detection of GCs in routine haematoxylin and eosin (H&E)‐stained sections can be challenging because small GCs may be overlooked and distinction between GCs and lymphoepithelial lesions may be difficult (Fisher et al., 2017; Haacke et al., 2018). Therefore, immunohistochemical identification using antibodies directed against CD21, expressed by FDCs (but also by B cells) or Bcl6, a transcription factor highly expressed by GC‐B cells, has been used (Bombardieri et al., 2007; Carubbi et al., 2019; Haacke et al., 2017), but consensus criteria regarding identification of GCs are lacking (Haacke et al., 2018). Hence, the aim of this study was to asses which staining is most suitable to unequivocally identify GCs in diagnostic salivary gland biopsies of pSS patients by comparing H&E, CD21 and Bcl6 stainings.

In our study, we restricted ourselves to these three markers, which can be easily applied in an automated fashion in diagnostic pathology laboratories. For this reason, we did not consider staining for other GC‐associated markers, such as activation‐induced deaminase, as potential candidates for identification of GCs in biopsies.

From 42 pSS patients, classified according to American College of Rheumatology (ACR)‐European League Against Rheumatism (EULAR) classification criteria (Shiboski et al., 2017), both a labial salivary gland and a parotid salivary gland biopsy were obtained (see Table 1). Four‐micrometre‐thick serial sections of salivary gland biopsies were stained with H&E and immunohistochemically for CD21 and Bcl6. For detailed ethical approval information, staining characteristics and statistical analysis see Appendix S1.

**Table 1:** Demographic, clinical and histological parameters of patients with primary Sjögren's syndrome

	pSS patients (*n* = 42)
Demographic characteristics	
Age, years	52 ± 13
Female, *n* (%)	41 (97.6)
Caucasian, *n* (%)	41 (97.6)
Serological parameters	
RF positive, *n* (%)	25 (59.5)
ANA positive, *n* (%)	10 (23.8)
Anti‐SSA positive, *n* (%)	32 (76.2)
Anti‐SSB positive, *n* (%)	15 (35.7)
IgG	15.4 [11.7–19.4]
ESR	23.0 [9.8–45.5]
CRP	2.8 [1.0–5.5]
Clinical parameters	
ESSDAI score	3.5 [2.0–9.0]
Schirmer, mm/5 min	2.5 [0.0–5.0]
UWS, ml/min	0.1 [0.0–0.2]
Histopathological parameters of the labial gland	
Focus score	1.3 [1.0–2.4]
≤70% IgA plasma cells, *n* (%)	19 (45.2)
Lymphoepithelial lesions, *n* (%)	16 (38.1)
Relative area of CD45^+^ infiltrate*	9.1 [6.1–19.8]
Histopathological parameters of the parotid gland	
Focus score	1.0 [0.0–1.7]
≤70% IgA plasma cells, *n* (%)	12 (28.6)
Lymphoepithelial lesions, *n* (%)	18 (42.9)
Relative area of CD45^+^ infiltrate*	4.5 [1.4–17.0]

Note

Patients were classified according to the ACR‐EULAR criteria. Data are represented as mean ± *SD*, median [95% CI] or number (%).

Abbreviations: ANA, antinuclear antibodies; CRP, C‐reactive protein; ESR, erythrocyte sedimentation rate; ESSDAI, European League Against Rheumatism SS Disease Activity Index; *n*, number of patients; pSS, primary Sjögren's syndrome; RF, rheumatoid factor; SSA, Sjögren's syndrome antigen A; SSB, Sjögren's syndrome antigen B; UWS, unstimulated whole saliva.

* % of area of lymphocytic infiltrate in salivary gland parenchyma (Aperio ImageScope v12.0).

In H&E‐stained sections, a GC was defined as a clearly visible lighter area in a lymphocytic infiltrate containing centrocytes, centroblasts, FDCs and macrophages. In CD21‐stained sections, a network of positive staining within a focus was classified as a FDC network. In Bcl6‐stained sections, a cluster of ≥5 adjacent positive cells within a focus was classified as a GC (Haacke et al., 2017). Even though Bcl6 is also expressed by follicular helper T cells, this expression does not interfere with detection of GCs as these cells are not organised in clusters as GCs, but lie scattered throughout the tissue (Figure 1).

**Figure 1:**
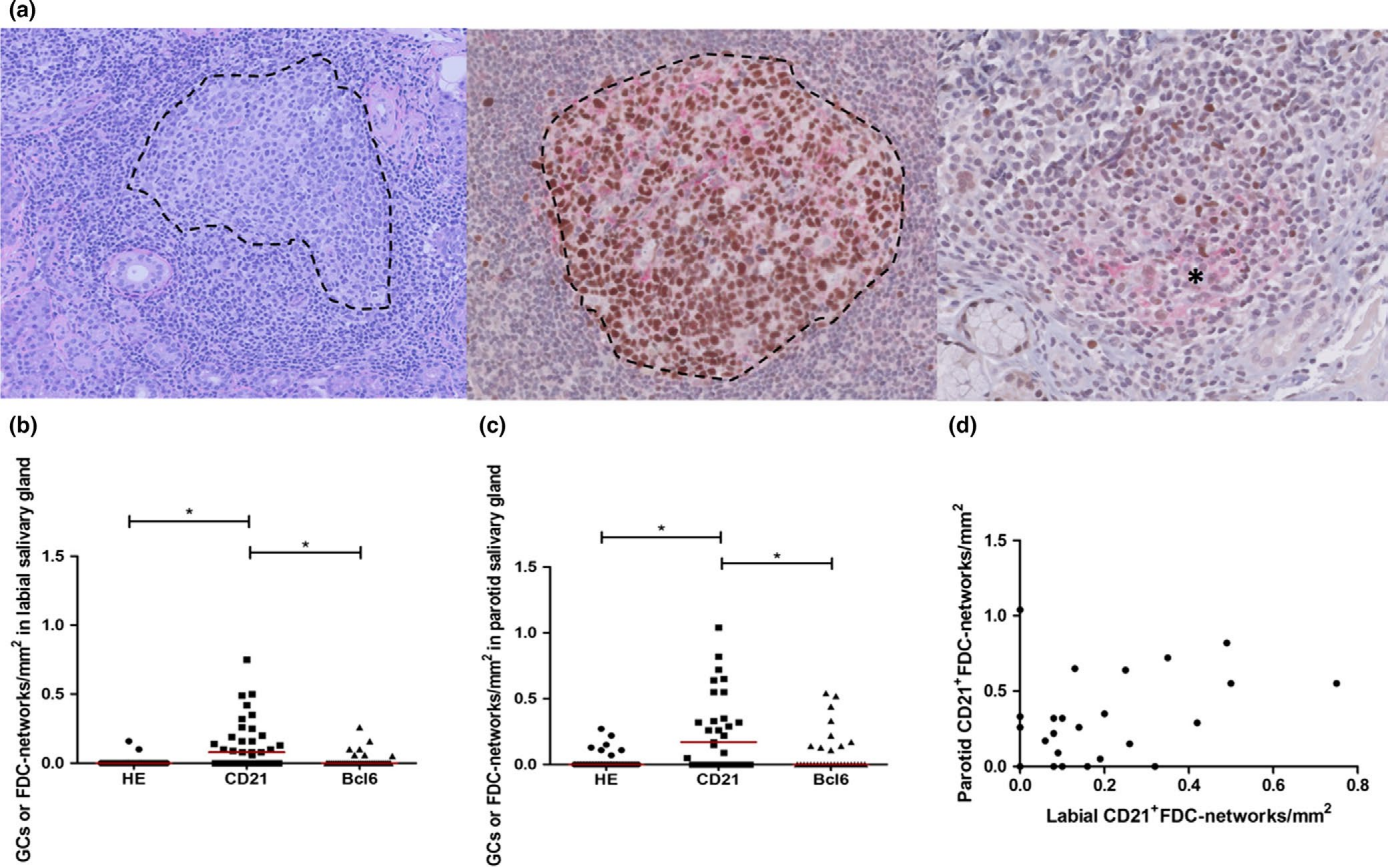
Presence of germinal centres and follicular dendritic cell networks in salivary gland biopsies of patients with primary Sjögren's syndrome. (a) Histopathological identification of germinal centres. Paraffin‐embedded parotid gland biopsy of a patient with primary Sjögren's syndrome stained with H&E (left panel) and by double immunohistochemistry for CD21 (red) and Bcl6 (brown) (middle and right panel). The left panel shows a periductal focus with a H&E‐stained GC (indicated by a dotted line, magnification 10×); the middle panel a CD21^+^FDC network with a Bcl6^+^GC (indicated by a dotted line, magnification 20×); and the right panel a CD21^+^FDC network (indicated by an asterisk, magnification 20×) without a GC. (b) Number of GCs or FDC networks/mm^2^ in labial (*n* = 36) salivary gland tissue after staining with H&E and immunohistochemically for Bcl6 or CD21. (c) Number of GCs or FDC networks/mm^2^ parotid (*n* = 31) salivary gland tissue after staining with H&E and immunohistochemically for Bcl6 or CD21. (d) Spearman's rank‐order correlation revealed a significant positive association between CD21^+^FDC networks/mm^2^ in parotid and labial salivary gland biopsies (Figure 1d *r* = .60, *p* = .001). Red lines indicate median values, *p < .05

Six labial and eleven parotid salivary gland biopsies did not contain any H&E‐defined periductal foci. For the remaining biopsies, 36 labial and 31 parotid glands, all individual H&E‐defined foci (210 labial and 141 parotid glands) were analysed on serial sections. This staining revealed that 1% (3/210) of labial gland foci and 6% (9/141) of parotid gland foci contained H&E‐defined GCs. Immunohistochemical staining for CD21 revealed that 24% (50/210) of the foci in the labial gland and 49% (69/141) of the foci in the parotid gland contained CD21^+^FDC networks (Table 2). Importantly, after staining for Bcl6, we showed that only 18% (9/50) of the labial gland foci with CD21^+^FDC networks and 32% (22/69) of the parotid gland foci with CD21^+^FDC networks also comprised Bcl6^+^GCs. Apparently, not all foci contain CD21^+^FDC networks and not all foci with CD21^+^FDC networks also harbour Bcl6^+^GCs. This was confirmed by dual CD21/Bcl6 staining (Figure 1a). Consequently, the number of CD21^+^FDC networks/mm^2^ was significantly higher than the number of H&E^+^‐ and Bcl6^+^‐defined GCs/mm^2^ in both labial and parotid salivary glands (Figure 1b and c). We observed a significant correlation between CD21^+^FDC networks/mm^2^ in parotid and labial salivary gland biopsies (Figure 1d, *r* = .60, *p* = .001), indicating comparability in lymphoid organisation at these two anatomical sites. Such a correlation was not seen for the presence of H&E‐ or Bcl6‐defined GCs.

**Table 2:** Comparison between the number of germinal centres in labial and parotid salivary gland biopsies of primary Sjögren's syndrome patients

	Labial salivary gland	Parotid salivary gland
Number of biopsies with foci	36	31
Number of foci	210	141
% H&E^+^GCs	1.4 (3/210)	6.4 (9/141)
% Bcl6^+^GCs	4.3 (9/210)	15.6 (22/141)
% CD21^+^FDC networks	23.8 (50/210)	48.9 (69/141)

Abbreviations: FDC, follicular dendritic cell; GCs, germinal centres; H&E, haematoxylin and eosin.

In a recent study, Carubbi et al. analysed the usage of CD3/CD20 as well as CD21 and Bcl6 as markers for the detection of GCs (Carubbi et al., 2019). While they conclude that combination of CD3/CD20 and CD21 should be recommended for assessment of GCs, we clearly show here that usage of CD21 as surrogate marker for GCs significantly overestimates GC counts. The reason for this is that formation of B‐cell follicles and presence of CD21^+^FDC networks (which are also present in primary B‐cell follicles) do not also imply presence of GCs (MacLennan, 1994). On the other hand, staining with H&E revealed fewer GCs compared to staining for Bcl6, most likely because small GCs can easily be overlooked on H&E.

Although staining for CD21 is thus less appropriate for detection of GCs, staining for CD21 is still valuable. FDCs play an essential role in the spatial orientation and B/T‐cell compartmentalisation in ectopic lymphoid tissues due to their CXCL13 producing property. Presence of FDC networks suggests a more advanced stage of ectopic lymphoid development and may therefore be a useful marker for classification of the organisation of glandular tissue (Jonsson & Skarstein, 2008).

In conclusion, we propose to use Bcl6 as a simple, sensitive and specific marker for unequivocal identification of GCs in salivary gland biopsies of (suspected) pSS patients. Large prospective studies are now needed to evaluate whether presence of GCs in diagnostic salivary gland biopsies for pSS is a risk factor for non‐Hodgkin lymphomas or not, and whether it can be used for stratification of pSS patients for personalised medicine (Delli et al., 2019; Kroese et al., 2018).

## CONFLICT OF INTEREST

There are no competing interests for any author.

## AUTHOR CONTRIBUTIONS

UN, EAH, FGMK, BvdV, HB and AV were involved in study concept and design. HB and AV recruited patients. FKLS performed all salivary gland biopsies. UN, EAH and BvdV collected data. UN, EAH, FGMK, BvdV, AV, FKLS and HB analysed and interpreted the data. All authors critically reviewed the manuscript and approved the final version to be published.

## References

[odi13276-bib-0001] Bombardieri, M. , Barone, F. , Humby, F. , Kelly, S. , McGurk, M. , Morgan, P. , … Pitzalis, C. (2007). Activation‐induced cytidine deaminase expression in follicular dendritic cell networks and interfollicular large B cells supports functionality of ectopic lymphoid neogenesis in autoimmune sialoadenitis and MALT lymphoma in Sjögren's syndrome. Journal of Immunology, 179(7), 4929–4938. 10.4049/jimmunol.179.7.4929 17878393

[odi13276-bib-0002] Carubbi, F. , Alunno, A. , Cipriani, P. , Coletti, G. , Bigerna, B. , Manetti, M. , … Gerli, R. (2019). Different operators and histologic techniques in the assessment of germinal center‐like structures in primary Sjögren's syndrome minor salivary glands. PLoS ONE, 14(1), e0211142 10.1371/journal.pone.0211142 30682150 PMC6347225

[odi13276-bib-0003] Delli, K. , Villa, A. , Farah, C. S. , Celentano, A. , Ojeda, D. , Peterson, D. E. , … Vissink, A. (2019). World workshop on Oral Medicine VII: Biomarkers predicting lymphoma in the salivary glands of patients with Sjögren's syndrome – a systematic review. Oral Diseases, 25(S1), 49–63. 10.1111/odi.13041 30663837

[odi13276-bib-0004] Fisher, B. A. , Jonsson, R. , Daniels, T. , Bombardieri, M. , Brown, R. M. , Morgan, P. , … Barone, F. (2017). Standardisation of labial salivary gland histopathology in clinical trials in primary Sjögren's syndrome. Annals of Rheumatic Diseases, 76(7), 1161–1168. 10.1136/annrheumdis-2016-210448 PMC5530351 27965259

[odi13276-bib-0005] Fox, R. I. (2017). Standardisation of labial salivary gland biopsies in Sjögren's syndrome: Importance for the practicing rheumatologist. Annals of Rheumatic Diseases, 76, 1159–1160. 10.1136/annrheumdis-2016-210851 28254788

[odi13276-bib-0006] Haacke, E. A. , Van Der Vegt, B. , Vissink, A. , Spijkervet, F. K. L. , Bootsma, H. , & Kroese, F. G. M. (2017). Germinal centres in diagnostic labial gland biopsies of patients with primary Sjogren's syndrome are not predictive for parotid MALT lymphoma development. Annals of Rheumatic Diseases, 76(10), 1781–1784. 10.1136/annrheumdis-2017-211290 28710097

[odi13276-bib-0007] Haacke, E. A. , van der Vegt, B. , Vissink, A. , Spijkervet, F. K. L. , Bootsma, H. , & Kroese, F. G. M. (2018). Standardisation of the detection of germinal centres in salivary gland biopsies of patients with primary Sjögren's syndrome is needed to assess their clinical relevance. Annals of Rheumatic Diseases, 77(6), e32 10.1136/annrheumdis-2017-212164 28939628

[odi13276-bib-0008] Haacke, E. A. , van der Vegt, B. , Vissink, A. , Spijkervet, F. K. L. , Bootsma, H. , Kroese, F. G. (2019). Germinal centers in diagnostics biopsies of patients with primary Sjögren's syndrome are not a risk factor for non‐Hodgkin's lymphoma but a reflection of high disease activity: comment on the article by Sène et al Arthritis & Rheumatology, 71, 170–171. 10.1002/art.40715 30178631

[odi13276-bib-0009] Jonsson, M. V. , & Skarstein, K. (2008). Follicular dendritic cells confirm lymphoid organization in the minor salivary glands of primary Sjögren's syndrome. Journal of Oral Pathology & Medicine, 37, 515–521. 10.1111/j.1600-0714.2008.00674.x 18662248

[odi13276-bib-0010] Kroese, F. G. M. , Abdulahad, W. H. , Haacke, E. , Bos, N. A. , Vissink, A. , & Bootsma, H. (2014). B‐cell hyperactivity in primary Sjögren's syndrome. Expert Review of Clinical Immunology, 10, 483–499. 10.1586/1744666X.2014.891439 24564507

[odi13276-bib-0011] Kroese, F. G. M. , Haacke, E. , & Bombardieri, S. (2018). The role of salivary gland histopathology in primary Sjögren's syndrome: Promises and pitfalls. Clinical and Experimental Rheumatology, 36, 222–2233. 30156550

[odi13276-bib-0012] Le Pottier, L. , Devouchelle, V. , Fautrel, A. , Daridon, C. , Saraux, A. , Youinou, P. , & Pers, J. O. (2009). Ectopic germinal centers are rare in Sjögren's syndrome salivary glands and do not exclude autoreactive B cells. Journal of Immunology, 182, 3540–3547. 10.4049/jimmunol.0803588 19265132

[odi13276-bib-0013] MacLennan, I. (1994). Germinal centers. Annual Review of Immunology, 12, 117–139. 10.1146/annurev.iy.12.040194.001001 8011279

[odi13276-bib-0014] Muramatsu, M. , Kinoshita, K. , Fagarasan, S. , Yamada, S. , Shinkai, Y. , & Honjo, T. (2000). Class switch recombination and hypermutation require activation‐induced cytidine deaminase (AID), a potential RNA editing enzyme. Cell, 102(5), 553–563. 10.1016/S0092-8674(00)00078-7 11007474

[odi13276-bib-0015] Nishishinya, M. B. , Pereda, C. A. , Muñoz‐Fernández, S. , Pego‐Reigosa, J. M. , Rúa‐Figueroa, I. , Andreu, J.‐L. , … Loza Santamaría, E. (2015). Identification of lymphoma predictors in patients with primary Sjögren's syndrome: A systematic literature review and meta‐analysis. Rheumatology International, 35(1), 17–26. 10.1007/s00296-014-3051-x 24899571

[odi13276-bib-0016] Risselada, A. P. , Looije, M. F. , Kruize, A. A. , Bijlsma, J. W. , & van Roon, J. A. (2013). The role of ectopic germinal centers in the immunopathology of primary Sjögren's syndrome: A systematic review. Seminars in Arthritis and Rheumatology, 42(4), 368–376. 10.1016/j.semarthrit.2012.07.003 22995442

[odi13276-bib-0017] Salomonsson, S. , Jonsson, M. V. , Skarstein, K. , Brokstad, K. A. , Hjelmström, P. , Wahren‐Herlenius, M. , & Jonsson, R. (2003). Cellular basis of ectopic germinal center formation and autoantibody production in the target organ of patients with Sjögren's Syndrome. Arthritis & Rheumatology, 48, 3187–3201. 10.1002/art.11311 14613282

[odi13276-bib-0018] Sène, D. , Ismael, S. , Forien, M. , Charlotte, F. , Kaci, R. , Cacoub, P. , … Lioté, F. (2018). Ectopic germinal center‐like structures in minor salivary gland biopsy tissue predict lymphoma occurrence in patients with primary Sjögren's syndrome. Arthritis & Rheumatology, 70(9), 1481–1488. 10.1002/art.40528 29669392

[odi13276-bib-0019] Shiboski, C. H. , Shiboski, S. C. , Seror, R. , Criswell, L. A. , Labetoulle, M. , Lietman, T. M. , … Wu, A. (2017). 2016 american college of rheumatology/European league against rheumatism classification criteria for primary Sjögren's syndrome: a consensus and data‐driven methodology involving three international patient cohorts. Arthritis & Rheumatology, 69(1), 35–45. 10.1002/art.39859 27785888 PMC5650478

[odi13276-bib-0020] Theander, E. , Vasaitis, L. , Baecklund, E. , Nordmark, G. , Warfvinge, G. , Liedholm, R. , … Jonsson, M. V. (2011). Lymphoid organisation in labial salivary gland biopsies is a possible predictor for the development of malignant lymphoma in primary Sjögren's syndrome. Annals of Rheumatic Diseases, 70(8), 1363–1368. 10.1136/ard.2010.144782 PMC3128323 21715359

