# Supplementary material for: Bcl6 for identification of germinal centres in salivary gland biopsies in primary Sjögren's syndrome
Source: Oral Dis. 2020 Jan 28;26(3):707–10. doi: 10.1111/odi.13276 (PMC7187171; doi:10.1111/odi.13276)
Supplement: Supplementary file 1 [file ODI-26-707-s001.docx]

**Supplementary material**

**Ethical approval information** The study was approved by the Medical Research Ethics Committee of the UMCG, the Netherlands (METc2013.066). All participants gave consent according to the declaration of Helsinki.

**Study information** Biopsies for this retrospective observational study were collected from 2014 until 2016. Inclusion and exclusion criteria are shown in supplementary table 1. All labial and parotid salivary gland biopsies were performed by one Oral and Maxillofacial surgeon (FKLS) at the University Medical Center Groningen.

**Immunohistological staining and histopathological assessment**

CD21 staining: Sections were deparaffinised and antigen retrieval was performed (EDTA, pH 8). Endogenous peroxidase activity was blocked using H_2_O_2_ and PBS. Slides were incubated with CD21 antibodies (2G9; Cell Marque Corporation, USA) for 75 min. After rinsing, sections were treated with horse radish peroxidase (HRP) polymer (goat anti-mouse IgG) for 40 min. Staining was visualised with DAB and the sections were counterstained with haemotoxylin. This staining procedure was optimized for the detection of FDC networks.

Bcl6 staining and CD21/Bcl6 double staining: Bcl-6 (GI19E/A8; Ventana Medical systems, USA, prediluted by supplier) staining and CD21/Bcl6 double staining was performed after deparaffinisation, pre-treatment with Ultra CC1 (Ventana Medical Systems, USA), antigen retrieval and endogenous peroxidase blocking using the Benchmark automated staining platform (Ventana Medical Systems, USA). The double staining was performed serially.

All foci in labial and parotid salivary gland parenchyma were analysed for the presence of CD21^+^FDC-networks and for H&E^+^- or Bcl6^+^GCs by a trained researcher (UN), an experienced pathology resident (EH) and a head and neck pathologist (BvdV). Discrepancies between observers were resolved in a consensus meeting.

**Statistical analysis**

Data were analysed using SPSS version 23 statistical software (SPSS Inc., Chicago, IL). Differences between groups were tested with Mann-Whitney U test. Correlation analysis was performed using the Spearman’s rank order correlation. *P*-values <0.05 were considered statistically significant.

Supplementary Table 1. Inclusion and exclusion criteria

| Inclusion criteria | Exclusion criteria |
| --- | --- |
| - Ability to give informed consent. - Male or female patients 18 years of age or older. - Patients, classified according to ACR-EULAR classification criteria. - Must be willing to have a standard physical exam as part of standard clinical care and a complete diagnostic work-up according to the ACR criteria for ocular staining, labial salivary gland biopsy and serology. - Must be willing to have a standard physical exam and complete AECG diagnostic tests as part of standard clinical care (including eye exam, oral exam, salivary gland exam and biopsy). - Must be willing to donate 1ml of stimulated, whole saliva in 30 minutes or less. If a participant cannot produce 1ml during a 30 min collection period, subject will be unevaluable and will be considered a screen failure and withdrawn from the study. - Subjects must be willing to have a labial salivary gland biopsy in addition to a parotid biopsy. - Must be willing and able to give approximately 8ml of blood. | - Previous radiation to the head and neck. - Confirmed hepatitis C virus infection, which may cause SS-like signs and symptoms. - Known HIV infection, which can cause salivary gland infiltrates and enlargements similar to SS. - Sarcoidosis, which may cause alike signs and symptoms. - Graft-versus-host disease, which may cause SS-like signs and symptoms. - Oral cancer or history of oral cancer. - Presence of MALT lymphoma. - Pregnancy based on self-report. - Previously confirmed diagnosis of autoimmune disease known to be associated with sSS (RA, SLE, CREST, scleroderma, mixed connective tissue disease, polymyositis). - Insufficient biopsy material harvested from either labial or parotid salivary gland. - Absence of any focus in the labial and parotid gland biopsy. |

*Note*

Abbreviations: ACR, American College of Rheumatology; AECG, American-European Consensus Group; EULAR, European League Against Rheumatism; CREST, Calcinosis, Raynaud’s syndrome, Esophageal dysmotility, Sclerodactylyl, Telangiectasia; MALT, Mucosa associated lymphoid tissue; RA, Rheumatoid arthritis; SLE, Systemic lupus erythematosus; sSS, secondary Sjögren’s syndrome.
